# YouTube online videos as a source for patient education of cervical spondylosis—a reliability and quality analysis

**DOI:** 10.1186/s12889-023-16495-w

**Published:** 2023-09-20

**Authors:** Hong Wang, Chunyi Yan, Tingkui Wu, Xiang Zhang, Junbo He, Zhihao Liu, Hao Liu

**Affiliations:** Department of Orthopedics, West China Hospital, Sichuan University, #37 Guoxue Alley, Wuhou District, Chengdu, Sichuan China

**Keywords:** Cervical spondylosis, Patient education, Online videos, YouTube, Reliability, Quality

## Abstract

**Background:**

Given a prolonged course of Cervical spondylosis (CS) could cause irreversible neurological deficits, it is crucial to disseminate CS-related health information to the public to promote early diagnosis and treatment. YouTube has been widely used to search for medical information. However, the reliability and quality of videos on YouTube vary greatly. Thus, this study aimed to assess the reliability and educational quality of YouTube videos concerning CS and further explore strategies for optimization of patient education.

**Methods:**

We searched YouTube online library for the keywords “cervical spondylosis”, “cervical radiculopathy” and “cervical myelopathy” on January 15, 2023. Ranked by “relevance”, the first 50 videos of each string were recorded. After exclusions, a total of 108 videos were included. All videos were extracted for characteristics and classified based on different sources or contents. Two raters independently evaluated the videos using Journal of American Medical Association (JAMA) benchmark criteria, Modified DISCERN (mDISCERN) tool, Global Quality Scale (GQS) and Cervical-Spondylosis-Specific Scale (CSSS), followed by statistical analyses. All continuous data were described as median (interquartile range).

**Results:**

All videos had median values for JAMA, mDISCERN, GQS and CSSS scores of were 3.00 (1.00), 3.00 (2.00), 2.00 (1.00) and 7.00 (8.88), respectively. There were significant differences in VPI (*P* = 0.009) and JAMA (*P* = 0.001), mDISCERN (*P* < 0.001), GQS (*P* < 0.001) and CSSS (*P* < 0.001) scores among different sources. Videos from academic source had advantages in reliability and quality scores than other sources. VPI (*P* < 0.001), mDISCERN (*P* = 0.001), GQS (*P* < 0.001) and CSSS (*P* = 0.001) scores also significantly differed among videos of various contents. Spearman correlation analysis indicated VPI was not correlated with either reliability or quality. Multiple linear regression analysis showed a longer duration and an academic source were independent predictors of higher reliability and quality, while a clinical source also led to the higher video quality.

**Conclusions:**

The reliability and educational quality of current CS-related videos on YouTube are unsatisfactory. Users face a high risk of encountering inaccurate and misleading information when searching for CS on YouTube. Longer duration, source of academic or clinician were closely correlated to higher video reliability and quality. Improving the holistic reliability and quality of online information requires the concerted effort from multiple parties, including uploaders, the platform and viewers.

## Background

Cervical spondylosis (CS) is a chronic and progressive degenerative process of the cervical spine featuring the pathological change of vertebrae, joints, intervertebral discs, and other relevant structures. [1] As the common cause of neurological dysfunction in adults, CS may progress into cervical radiculopathy (CR) and cervical myelopathy (CM), with principal symptoms of pain, paresthesia and muscle weakness in the neck and/or extremities. [2, 3] In severe cases, additional manifestations such as bladder problems, ataxia, restricted motion, and even paralysis may ensue, portending poor outcomes. [4] Also, CS could be simultaneous with several comorbidities of depression, anxiety and sleep disorders etc. [5–7] Due to demographic aging and shifting lifestyle patterns, the prevalence of symptomatic cervical spondylosis is increasing, with up to 13.76% reported by a community-based study. Furthermore, onset is occurring at increasingly younger ages. [8] This has engendered a hefty burden on global economics and productivity, emerging as a salient public health challenge worldwide. [9, 10].

Given that the protracted course of CS could result in an irreversible detrimental outcome, [1, 4] early diagnosis and appropriate management are essential to obviate lifelong disability. However, the information asymmetry between doctors and general public perturbs patients aware of their conditions. Therefore, it is imperative to provide precise health education about CS to the public.

With the development of the internet and the exponential growth of digital information, an increasing number of netizens choose to consult and seek disease-related information online. [11, 12] YouTube, as the second most visited and the most popular video sharing site, has more than 22.8 billion visits per month and yields videos about specific medical information and tutorials that contain potential applicability for patient education and even management. [13] However, since the lack of the regulation, the quality of video information from YouTube is uneven [14, 15], especially concerning medical field. Substandard, inadequate or erroneous information diverges from professional clinical advice, engendering wrong perceptions in patients and leading to inappropriate self-assessment of their conditions. [16, 17] This may interfere with proper medical management for individuals and result in disease deterioration. For society as a whole, it could aggravate the doctor-patient relationship.

Hitherto, to the best of our knowledge, no research has quantified the reliability and quality of CS-related information available on YouTube. Herein, this study aims to assess the reliability and educational quality of YouTube videos regarding CS, and analyze relevant influencing factors, furtherly exploring strategies to optimize the quality of online health resources and guide patients to obtain valuable medical information related to CS.

## Methods

### Ethics approval and information consent

Since data from YouTube was all publicly available and no patients were involved in this study, the ethics committee approval and information consent were not required.

### Search strategy and video characteristics

**Fig. 1:**
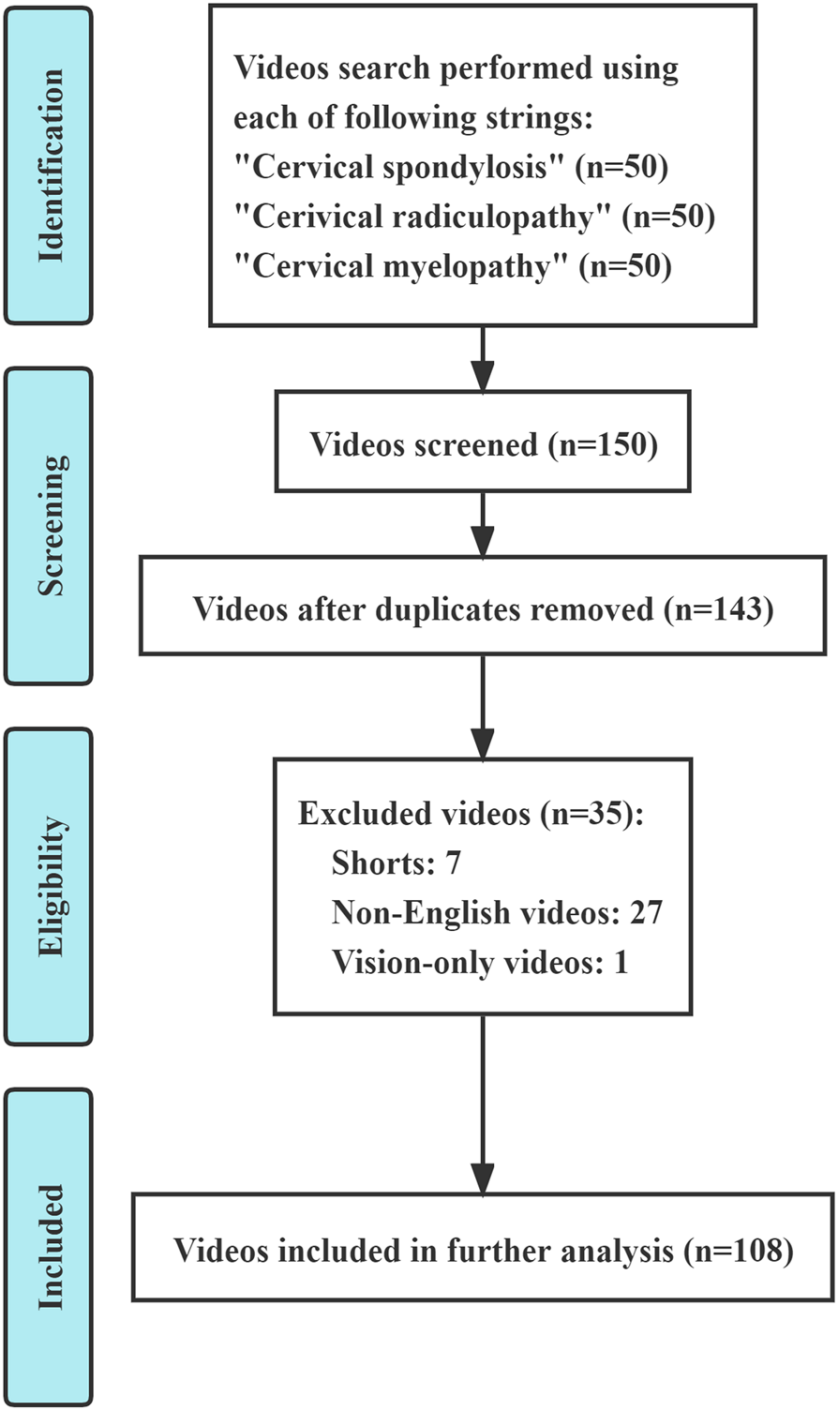
Flowchart of videos selection on YouTube

To evaluate the reliability and quality of online videos, we standardly searched for the keywords “cervical spondylosis”, “cervical radiculopathy” and “cervical myelopathy” on YouTube online library (https://www.youtube.com, January 15, 2023). Before conducting the search, we created an exclusive account and cleared browser cache and search history, also turning off data recording to avoid potential influence on the results. By default settings and ranking of “relevance”, we preliminarily recorded the first 50 videos in results of each string. This strategy could simulate the common browsing habit in most viewers and had been reported feasible in previous literatures. [18, 19] Furthermore, duplicate, irrelevant, non-English, audio-only, vision-only video, shorts or video with unacceptable audio/visual quality was excluded in this study. Finally, 108 videos were included for following analysis (Fig. 1).

The following data of video characteristics were extracted: (1) Title and URL, (2) uploader, (3) date of publication, (4) time since uploaded (till January 15, 2023), (5) video duration, (6) number of views, (7) number of likes, (8) number of dislikes, (9) number of referrers, (10) number of comments, (11) view ratio (views/day), (12) like ratio (like × 100/(like + dislike)), (13) uploader verification, (14) country/region of origin, (15) total views of host channel, (16) sum of subscribers of host channel and (17) Video Power Index (VPI). The specific calculation of VPI was as follows: view ratio × like ratio/100. VPI was an objective index to quantify the video popularity and had been applied in previous research. [20–22] Some of the indexes were collected through the reliable third-party browser plugin. [23, 24].

### Classification of video sources and contents

In accordance to previous studies [12, 25] and the actual searching results, the videos were sorted into six categories based on the source: (1) academic (uploaders affiliated with universities, colleges or research groups); (2) clinician (individual clinician or clinician groups without affiliation of academic institutions); (3) non-clinician (allied health workers other than licensed clinicians: physiotherapists etc.); (4) trainer; (5) medical source (health-related channels or websites) or (6) commercial source (corporations or for-profit organizations).

The videos were also classified into the following categories based on the content: (1) exercise training (exercise related to CS); (2) CS-specific information (pathophysiology, examinations, diagnosis etc.); (3) surgical technique; (4) non-surgical management; (5) advertisement. A single video was limited to one theme. If a video involved several content topics, the content that occupied the largest proportion or which viewers gained most from the video would be determined.

### Evaluation of video reliability and educational quality

Journal of American Medical Association (JAMA) benchmark criteria (Table 1), proposed by Silberg et al. [26], were used to evaluate the information reliability of included videos. Each of the four core standards (authorship, attribution, disclosure, and currency) is assigned one point, and the total JAMA score is calculated by summing up the fulfilled criteria. A maximal score of four represents the highest accuracy and reliability, whereas a score of zero indicates poor accuracy and reliability. Additionally, DISCERN tool, which was originally proposed by Charnock et al. and modified by singh et al., was adopted to verify the video reliability from another perspective (Table 2). [27, 28] Modified DISCERN (mDISCERN) tool is based on five binary yes/no questions, with every positive answer gaining one point and a maximal score of five indicating high reliability. Global Quality Scale (GQS) [29] was utilized for non-specific evaluation of videos’ educational quality (Table 3). GQS is a five-grade scale that ranges from one to five grades, with higher grades standing for higher quality.

**Table 1:** Journal of American Medical Association (JAMA) benchmark criteria

Criterion	Description
Authorship	Authors and contributors, their affiliations, and relevant credentials should be provided
Attribution	References and sources for all content should be listed clearly, and all relevant copyright information noted
Disclosure	"Ownership", sponsorship, advertising, underwriting, commercial funding arrangements or support, or potential conflicts of interest should be prominently and fully disclosed
Currency	Dates that content was posted and updated should be indicated

**Table 2:** Modified DISCERN (mDISCERN) criteria

Item	Description
1	Are the aims clear and achieved?
2	Are reliable sources of information used? (i.e., publication cited; provided by certified orthopedists or neurosurgeons)
3	Is the information presented balanced and unbiased?
4	Are additional sources of information listed for patient reference?
5	Are areas of uncertainty mentioned?

**Table 3:** Global quality scale (GQS) criteria

Score	Description
1	Poor quality and flow, most information missing; technique misleading; unlikely to be useful for patient education
2	Generally sparse quality and flow, some information provided but many important topics missing; technique poor; of very limited use to patients
3	Moderate quality and suboptimal flow, some important information provided adequately but others poorly discussed; technique basically adequate; somewhat useful for patients
4	Good quality and generally good flow, majority of information provided but some topics not covered; technique almost adequate; useful for patients
5	Excellent quality and flow, full information provided; technique adequate; highly useful for patients

There was no existing method to assess the educational content of CS videos specifically and comprehensively. Combining opinions from previous articles, reviews, guidelines and our clinical practice, we developed a novel scoring system entitled Cervical-Spondylosis-Specific Scale (CSSS). CSSS comprised four sections (information about CS, evaluation and diagnosis, treatment and postoperative course) and 19 sub-items in total. Diverse points were allocated to each item based on different priority and value. The total score was calculated by summing up the corresponding point(s) for all fulfilled items, with a maximum of 25 points indicating the highest educational quality for CS. Under this evaluation system, a high-quality CS-related video needs to elucidate the following information: the typical symptom of CS (neck pain, radiating pain, paresthesia etc.) and general nosogenesis (compression) and risk factors (age, poor postural habit, high loads); the main classification of CS (radiculopathy, myelopathy, etc.); diagnostic methods (physical examination, diagnostic imaging, differential diagnosis); treatment strategies (non-surgical and surgical options, highlight of the difference between treatments for CR and CM); posttreatment course (natural history, prognosis, complications). More specific items were shown in Table 4. Although CSSS was not validated, the similar structure for disease-specific scale had been broadly used for evaluation of video quality in peer-reviewed studies and been proven feasible. [30–32].

**Table 4:** Cervical-spondylosis-specific scale (CSSS) criteria

**Information about CS**
Describes symptoms: neck pain, radiating pain, stiffness, paresthesia (numbness, tingling, etc.), muscle weakness and dystonia (clumsy hands, gait abnormality, etc.), dysreflexia, restricted motion, ataxia, paralysis, bowel/bladder disturbance, etc. (*mentions 1–2 items: 1 point;* ≥ *3 items: 2 points*)
Describes epidemiology: prevalent in elders; male patients more than females (*0.5 point*)
Describes anatomy and/or function of cervical spine and relevant structures (*1 point*)
Describes mechanism and pathophysiology: caused by degenerative change of intervertebral disc and/or adjacent structures, leading to cervical nerve and/or vessels injured; physical compression and/or inflammation (*2 point*)
Mentions risk factors: age, trauma, poor postural habit, smoking, high loads, obesity, congenital factors, weak cervical muscles, etc. (*mentions 1–2 items: 1 point;* ≥ *3 items: 2 points*)
Discusses classification: radiculopathy, myelopathy, etc. (*1 point*)
**Evaluation and diagnosis**
Discusses physical and neurological examination: Spurling sign, shoulder abduction sign, Choi’s/tornado test, manual muscle exam, Eaton sign, Barre-Lieou sign, Hoffmann sign, Babinski sign, L’hermitte sign, finger-escape sign, Wartenberg sign, sensory exam, etc. (*mentions 1–2 items: 1 point;* ≥ *3 items: 2 points*)
Discusses diagnostic imaging: X-ray, CT, MRI (*mentions terms: 1 point; also provides example and explanation: 2 points*)?
Mentions EMG and/or PRO measures: VAS score, NDI score, mJOA score etc. (*1 point*)
Mentions differential diagnosis (*1 point*)
**Treatment**
Describes conservative treatments: first-line recommendation for symptomatic patients without myelopathy (*1 point*)
Mentions non-surgical options: cervical exercise, traction, pharmacotherapy, chiropractic, thermotherapy, electrotherapy, acupuncture, radiofrequency ablation, epidural steroid injection, neck collar immobilization, etc. (*mentions 1–2 items: 1 point;* ≥ *3 items: 2 points*)
Mentions medication: NSAIDs, neurotrophic drugs, muscle relaxants, steroids (*1 point*)
Mentions surgical treatment and indications: patients with severe/progressive pain or neurological deficits, refractory to conservative therapy (*1 point*)
Discusses different cervical surgical options with relevant anatomy: anterior or posterior, fusion or non-fusion, open or minimally invasive (*2 points*)
Discusses complications: Infection, implant-related complications, neurological deficits, vascular injury, CSF leakage, dysphagia, pseudoarthrosis, psychosocial implication, etc. (*1 point*)
**Posttreatment course**
Discusses natural history and/or prognosis: cervical radiculopathy is often self-limited; majority of patients improve after early and appropriate management; old age, long-term disease course, severe and irreversible symptoms, other underlying diseases, psychological symptoms indicate poor prognosis (*1 point*)
Mentions postoperative management: postoperative functional exercise and neurological rehabilitation under professional guidance as soon as possible (*1 point*)
Outlines timeline of functional recovery (*0.5 point*)

*CS* cervical spondylosis, *CT* computer tomography, *MRI* magnetic resonance imaging, *EMG* electromyography, *PRO* patient-reported outcome, *VAS* visual analogue scale, *NDI* neck disability index, *mJOA* modified Japanese Orthopedic Association, *NSAIDs* nonsteroidal anti-inflammatory drugs, *CSF* cerebrospinal fluid

Two independent orthopedic doctors (H.W., C.Y.) assessed all the included videos using above scoring systems and repeated once after two weeks. For a single video, both the video itself and its description were taken into account. The original results assessed by two doctors were recorded separately. Any discrepancy between both was arbitrated by the third reviewer (H.L.) to achieve a unanimous result.

### Statistical analysis

Video characteristics, reliability and educational quality were quantified by descriptive statistics. The missing data were processed using multiple Imputation. All continuous data in the study were described as median (interquartile range) as they didn’t comply normality. Qualitative data were expressed as fractions. Kruskal–Wallis test was utilized to evaluate intergroup differences of variables based on different video sources or contents, followed by post-hoc analyses of Bonferroni correction. Spearman correlation analysis was used to explore the correlation among VPI, JAMA, mDISCERN, GQS and CSSS scores. Then multiple linear regression analysis was applied to determine the independent predictor of the five above indexes from video characteristics, sources and contents. Variables that showed a univariate relationship (*P* < 0.20) with the target index or that were considered or reported relevant were incorporated into the regression model. Variables were carefully selected based on the number of events available to ensure the final model was parsimonious. [33] The categories of medical source and CS-specific information were set as the reference dummy variable of video sources and video contents, respectively, for the unified comparisons.

Intraobserver and interobserver agreements of four scoring systems (JAMA, mDISCERN, GQS, CSSS) were appraised by intraclass correlation coefficient (ICC), respectively. ICC values were interpreted referring to the guidelines: excellent (> 0.90), good (0.75–0.90), moderate (0.50–0.75) and poor (< 0.50). [34].

All the statistical analyses were performed by IBM SPSS Statistics v.26 (IBM Corp., Armonk, New York, USA). All reported P values were two-sided, and the difference was considered statistically significant when the P value < 0.05.

## Results

### Video baseline characteristics

**Fig. 2:**
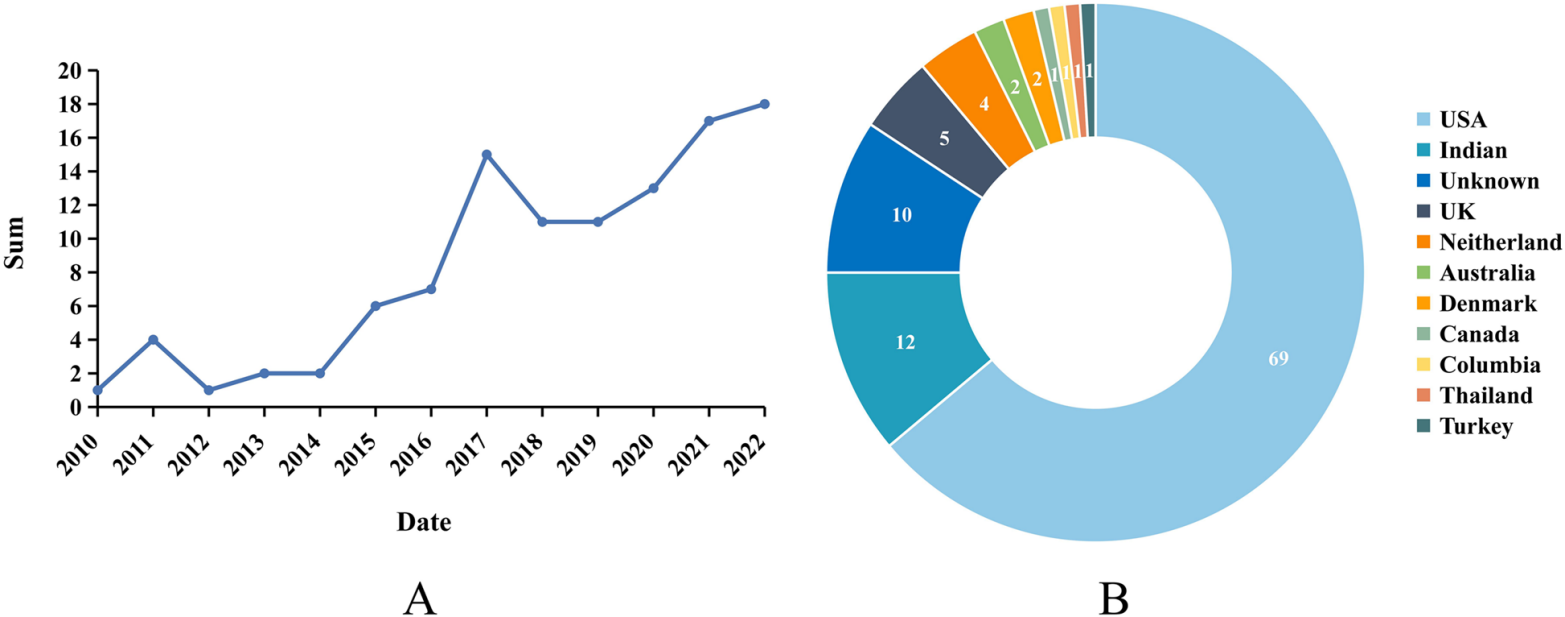
Video counts of each year from 2010 to 2022 (**A**); Distribution of videos based on original countries (**B**)

Of the 150 videos screened, 108 were eligible and included in the study. The earliest video was published on April 13, 2010. Among all included videos, the number of each year generally increased by time, and more than a half (54.63%, 59/108) were released in 2019–2022 (Fig. 2A). Excluding videos with unknown original country, the top three countries that produced the most videos were USA (69), Indian (12) and UK (5) (Fig. 2B). Through analysis of all videos, the median value of time since uploaded, video duration and view count of each video were 1329.00 (1629.00) days, 263.00 (511.00) seconds and 18,551.50 (62,456.00), respectively. Every video received the median likes and dislikes count of 201.00 (846.75) and 5.00 (26.75) with the median referrers of 0.00 (1.00) and the median comments of 16.00 (62.00). The median view ratio, like ratio and VPI were calculated by established formulas to be 19.20 (67.21), 97.99 (4.24) and 18.49 (67.06), respectively. Nineteen videos were shared by officially verified uploaders. Every video host channel has the median total views of 7,000,000.00 (37,348,325.00) and the median subscribers sum of 49,550.00 (337,895.00) (Table 5).

**Table 5:** The baseline characteristics and evaluation results of involved YouTube videos

Variable	Value
Sum of videos	108
Time since uploaded, days	1329.00 (1629.00)
Video duration, s	263.00 (511.00)
Number of views	18,551.50 (62,456.00)
Number of likes	201.00 (846.75)
Number of dislikes	5.00 (26.75)
Number of referrers	0.00 (1.00)
Number of comments	16.00 (62.00)
View ratio	19.20 (67.21)
Like ratio	97.99 (4.24)
Uploader verification, (Y/N)	19/89
Total views of host channel	7,000,000 (37,348,325)
Sum of subscribers to host channel	49,550.00 (337,895.00)
VPI	18.49 (67.06)
JAMA score	3.00 (1.00)
mDISCERN score	3.00 (2.00)
GQS score	2.00 (1.00)
CSSS score	7.00 (8.88)

*SD* standard deviation, *VPI* video power index, *JAMA* Journal of American Medical Association, *mDISCERN* modified DISCERN, *GQS* Global Quality Scale, *CSSS* Cervical-Spondylosis-Specific Scale

Continuous data were presented as median (interquartile range)

### Video sources and contents

**Fig. 3:**
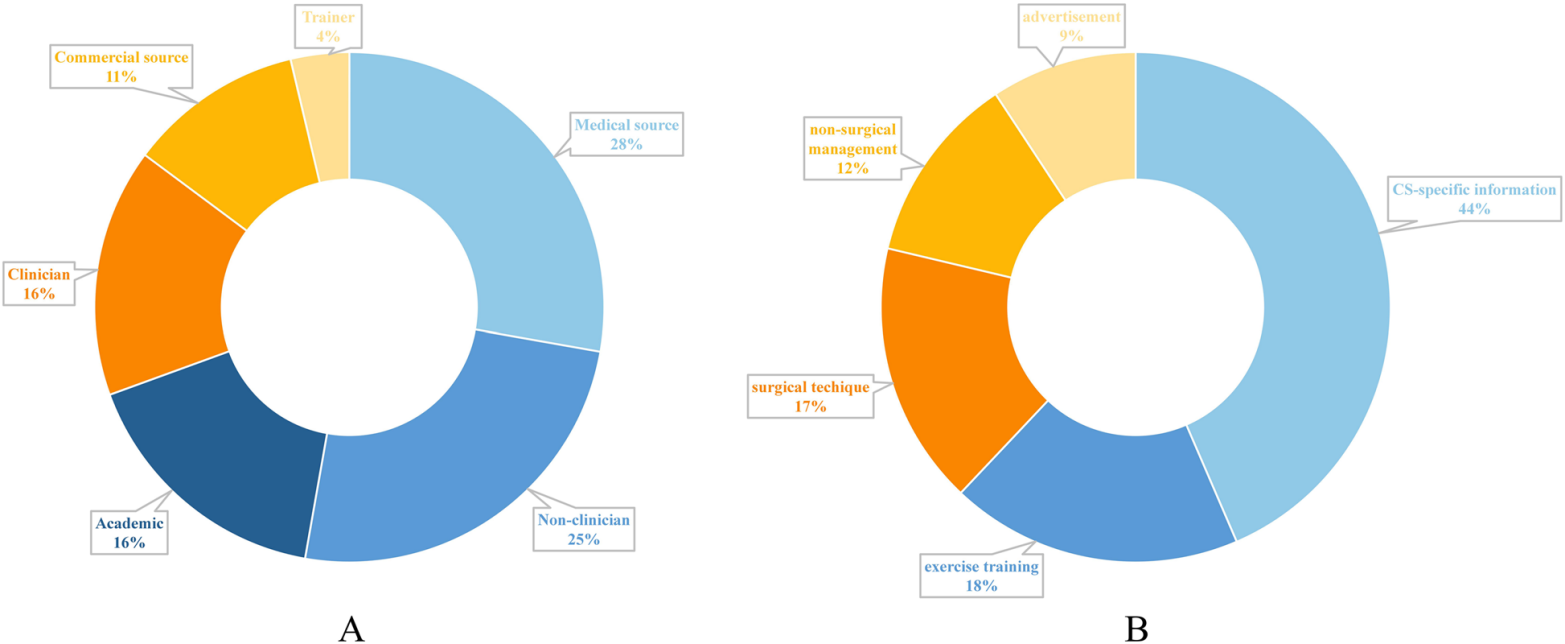
Categorical distribution of videos based on sources (**A**); Categorical distribution of videos based on contents (**B**)

All the videos were sorted into six sources: Medical source led the largest share (30/108, 28%), followed by non-clinician (27/108, 25%), academic (18/108, 17%), clinician (17/108, 16%), commercial source (12/108, 11%), and trainer (4/108, 3%). (Fig. 3A).

Among five categories based on video content, CS-specific information was the most frequently covered (47/108, 43%), followed by exercise training (20/108, 19%), surgical technique (18/108, 17%), non-surgical management (13/108, 12%), and advertisement (10/108, 9%) (Fig. 3B).

**Fig. 4:**
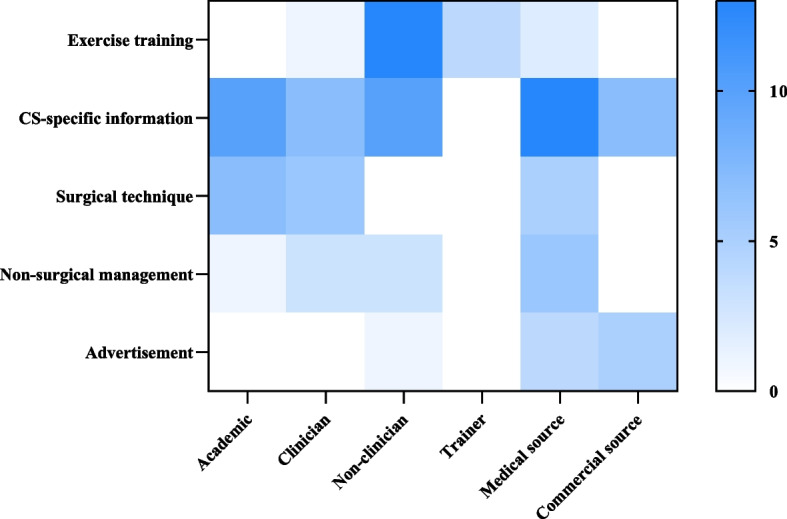
Heatmap of video counts concerning from different sources and contents

The heatmap provided an abstract representation of the number of videos with different content from different sources. Academics and clinicians focused more on CS-specific information and surgical techniques, while non-clinicians focused more on exercise training and CS-specific information. Trainers only produced videos about exercise training. Videos from medical source covered all topics, with a greater emphasis on CS-specific information. And videos from commercial source merely involved CS-specific information or non-surgical management (Fig. 4).

### Video reliability and educational quality

The median values for JAMA, mDISCERN, GQS and CSSS scores of all videos were 3.00 (1.00), 3.00 (2.00), 2.00 (1.00) and 7.00 (8.88), respectively (Table 5). The Intraobserver reliability within each rater was excellent for all four scales. Two raters achieved excellent agreement in JAMA scores (ICC: 0.906, 95% confidence interval (CI): 0.860–0.937), GQS scores (ICC: 0.927, 95% CI: 0.895–0.949), CSSS scores (ICC: 0.939, 95% CI: 0.908–0.960); and good agreement in mDISCERN scores (ICC: 0.888, 95% CI: 0.831–0.925).

There were significant differences in VPI (*P* = 0.009) and JAMA (*P* = 0.001), mDISCERN (*P* < 0.001), GQS (*P* < 0.001) and CSSS (*P* < 0.001) scores among different video uploading sources. The only significant discrepancy in VPI existed between videos from non-clinician and medical source (28.06 (111.34) vs. 12.34 (19.04), *P* < 0.05). Videos from academic source showed significant (*P* < 0.05) higher scores in the four scales than any other source, except for clinician source in GQS and CSSS (P > 0.05). Meanwhile, VPI (*P* < 0.001), mDISCERN (*P* = 0.001), GQS (*P* < 0.001) and CSSS (*P* = 0.001) scores significantly differed among videos of various contents. Videos about exercise training had higher VPI than those covering CS-specific information, surgical technique and non-surgical management with significance (*P* < 0.05). In contrast, the contents of surgical technique and non-surgical management led to significantly (*P* < 0.05) higher scores in mDISCERN, GQS and CSSS scales than exercise training. More detailed analytical results are displayed in Table 6.

**Table 6:** The median VPI values, median scores of reliability and quality of different video source or content

	**VPI**	**JAMA**	**mDISCERN**	**GQS**	**CSSS**
**Video source**					
Academic	20.00 (96.15)	3.00 (1.00)	4.00 (1.00)	4.00 (1.25)	14.50 (9.75)
Clinician	17.29 (76.48)	2.00 (1.00)	3.00 (1.50)	3.00 (2.00)	11.00 (10.00)
Non-clinician	28.06 (111.34)	3.00 (1.00)	3.00 (1.00)	2.00 (0.00)	5.00 (4.00)
Trainer	101.27 (56.84)	1.50 (1.75)	1.50 (1.00)	1.00 (0.75)	2.00 (3.00)
Medical source	12.34 (19.04)	2.00 (1.00)	3.00 (0.00)	2.00 (1.00)	6.00 (9.25)
Commercial source	11.06 (47.47)	2.00 (1.75)	2.00 (2.00)	2.00 (1.75)	6.50 (4.75)
*P* value	***0.009***	***0.001***	** < *0.001***	** < *0.001***	** < *0.001***
Significant differences in post-hoc tests^✝^	Non-clinician vs. medical source	Academic vs. clinician, non-clinician, trainer, medical source, commercial source	Academic vs. clinician, non-clinician, trainer, medical source, commercial source	Academic vs. non-clinician, trainer, medical source, commercial source; clinician vs. non-clinician, trainer	Academic vs. non-clinician, trainer, medical source, commercial source; clinician vs. non-clinician, trainer
**Video content**					
Exercise training	99.87 (170.16)	3.00 (1.00)	2.00 (1.00)	2.00 (1.75)	4.00 (5.00)
CS-specific information	19.95 (45.80)	2.00 (1.00)	3.00 (1.00)	2.00 (1.00)	7.00 (6.00)
Surgical technique	3.52 (13.89)	3.00 (2.00)	4.00 (2.25)	3.00 (2.00)	12.50 (13.25)
Non-surgical management	22.24 (69.35)	3.00 (1.00)	3.00 (1.50)	3.00 (2.00)	14.00 (13.25)
Advertisement	11.71 (12.97)	2.00 (1.00)	2.00 (2.00)	2.00 (1.00)	5.50 (3.50)
*P value*	** < *0.001***	***0.052***	***0.001***	** < *0.001***	***0.001***
Significant differences in post-hoc tests^✝^	Exercise training vs. CS-specific information, surgical technique, advertisement;		Surgical technique vs. exercise training, advertisement	Surgical technique vs. exercise training, advertisement; non-surgical management vs. exercise training, advertisement	Surgical technique and non-surgical management vs. exercise training

*VPI* video power index, *JAMA* Journal of American Medical Association, *mDISCERN* modified DISCERN, *GQS* Global Quality Scale, *CSSS* Cervical-Spondylosis-Specific Scale

Continuous data were presented as median (interquartile range)

^✝^Post hoc tests were performed using Bonferroni method

### Factors affecting video popularity, reliability and educational quality

Spearman correlation analysis revealed positive and significant correlations among every pair of JAMA, mDISCERN, GQS, and CSSS scores (*P* < 0.001 for each pair). However, none of these scores were significantly correlated with VPI (Table 7).

**Table 7:** Spearman correlation analysis between VPI, JAMA, mDISCERN, GQS and CSSS

		VPI	JAMA	mDISCERN	GQS	CSSS
VPI	CC	1.000	0.115	-0.040	0.113	0.034
	*P*	*-*	*0.238*	*0.678*	*0.243*	*0.728*
JAMA	CC	0.115	1.000	0.670	0.508	0.464
	*P*	*0.238*	*-*	**< *0.001***	**< *0.001***	**< *0.001***
mDISCERN	CC	-0.040	0.670	1.000	0.622	0.589
	*P*	*0.678*	**< *0.001***	*-*	**< *0.001***	**< *0.001***
GQS	CC	0.113	0.508	0.622	1.000	0.930
	*P*	*0.243*	** < *0.001***	** < *0.001***	*-*	** < *0.001***
CSSS	CC	0.034	0.464	0.589	0.930	1.000
	*P*	*0.728*	** < *0.001***	** < *0.001***	** < *0.001***	*-*

*CC* correlation coefficient, *VPI* video power index, *JAMA* Journal of American Medical Association, *mDISCERN* modified DISCERN *GQS* Global Quality Scale; *CSSS* Cervical-Spondylosis-Specific Scale

Multiple linear regression analysis showed that a higher VPI was correlated with a higher number of comments (*P* < 0.001), a verified uploader (*P* = 0.034), fewer subscribers to the host channel (*P* = 0.011). And compared to the CS-specific information, the content about exercise was a independent predictor to higher VPI (*P* = 0.005); A higher JAMA score was associated with longer video duration (*P* < 0.001), greater like ratio (*P* < 0.001), a verified uploader (*P* = 0.002). The videos from academic source were correlated to higher JAMA scores than medical source (*P* = 0.003); A higher mDISCERN score was closely related to longer video duration (*P* < 0.001), greater like ratio (*P* < 0.001). In comparison to medical source, the sources of academic (*P* < 0.001) and trainer (*P* = 0.001) were associated with higher and lower mDISCERN scores, respectively; A higher GQS score was correlated to longer video duration (*P* < 0.001). The sources of academic (*P* = 0.001) and clinician (*P* = 0.002) were independent predictors of higher GQS scores compared to medical source, and the contents about exercise training (*P* = 0.021) and advertisement (*P* = 0.009) were related to lower GQS scores than the CS-specific information; A higher CSSS score was in correlation with longer video duration (*P* < 0.001). And Compared to the medical source, the sources of academic (*P* = 0.005) and clinician (*P* = 0.006) were associated with higher CSSS scores while the sources of non-clinician (*P* = 0.024) and trainer (*P* = 0.033) were related with lower CSSS scores.

## Discussion

Given the neurological deficit caused by chronic CS course [1], it is crucial to promote early diagnosis and treatment. The internet provides another dimension to balance the information asymmetry between doctors and patients. Studies show that about 70–80% of netizens and 30% of orthopedic patients utilize the internet to acquire health information [35, 36], building their preliminary perception. With the advent of 5G technology, videos have emerged as a widely accepted medium for conveying information on the internet. This has led to the rise of numerous international visual websites, with YouTube being a prominent example. Unfortunately, the internet is replete with inaccurate and misleading information that can shape patients’ perspectives on their ailments in ways that are often at odds with professional recommendations, thereby reducing patient compliance. As clinicians, we may not be able to edit or correct all the public information shared on the web. However, we should at least understand the online information that patients receive, how it shapes their cognition, and how it can be optimized. This motivated the authors to undertake an exploration and evaluation of CS-related videos on YouTube.

The current results indicate that the reliability and educational quality of CS-related videos on YouTube are unsatisfactory, with the median JAMA, mDISCERN, GQS and CSSS scores of 3.00 (1.00), 3.00 (2.00), 2.00 (1.00) and 7.00 (8.88), respectively. This suggests that the common netizens or patients searching for information about CS on YouTube may be at a relatively high risk of encountering inadequate, inaccurate, or even misleading information. Our finding is consistent with previous research in the field of spinal health. Erdem et al. revealed the poor quality of videos covering kyphosis on YouTube, which had the mean JAMA, GQS and Kyphosis-Specific Scores (range: 0–32) of 1.36, 1.68, 3.02, respectively. [37] Similarly, Stogowski et al. studied 24 YouTube videos about anterior lumbar interbody fusion and concluded that the overall quality remains poor, with the mean DISCERN score of 38.21/75. [38] A detailed review about representative studies focusing on YouTube information of spine field was demonstrated in Table 8. In contrast, a few studies proposed a more positive attitude towards medical videos on YouTube. Unal-Ulutatar et al. searched for “systemic sclerosis” and “scleroderma” and determined 73% (84/115) of the videos were useful. [39] Ng et al. highlighted that there was an abundance of reliable and of high-quality YouTube videos with useful information on systemic lupus erythematosus. [40] The discrepancy in these conclusions may be attributed to the differences in studied fields and source proportions.

**Table 8:** Literature review of studies concerning YouTube information of spine fields

Author	Objective	Search strategy	Number of included videos and Classification strategy	Evaluation method	Key findings
Erdem et al. [37]	To confirm the accuracy and quality of the information in kyphosis videos shared on YouTube	Searched using keyword “kyphosis”; Selected first 50 videos in response to the query	50 •Based on the source: academic, physician, nonphysician, trainer, medical, patient, and commercial •Based on the content: exercise training, information about disease, patient experience, surgical technique, chiropractic treatment, and advertisement	JAMA score, GQS score, Kyphosis-Specific Score	•The quality of the information about kyphosis acquired on YouTube is poor. •Despite their high number of views, the information and accuracy of the videos uploaded by physicians were poor. •The low-quality videos were more readable, whereas videos with higher scores were not found “attractive” by the users
Ovenden et al. [41]	To assess the quality of anterior cervical discectomy and fusion videos available on YouTube and identify factors associated with video quality	Searched using phrase “anterior cervical discectomy and fusion”; Selected first 50 videos in response to the query	50 •Based on the source: patient testimonies, spinal surgeon, and other (paramedic companies, medical engineering companies, or media teams)	JAMA score, DISERN score, Health on the Net (HON) ranking systems	•The overall video quality was poor. •Surgeon authors’ videos scored higher than patient testimony videos when reviewed using the HON or JAMA systems.
Mohile et al. [42]	To evaluate YouTube’s current patient accessible health information on the topic of lumbar disc herniation (LDH)	Searched using 3 different strings: “disc herniation”, “lumbar disc herniation”, and “lower back disc herniation”; Selected first 50 videos in response to each query	77 •Based on the source: educational (physician), educational (non-physician), surgical Technique, advertisement, and patient testimonial. •Based on the content: diagnosis, treatment, and both	Diagnostic information assessment checklist for LDH, treatment information assessment checklist for LDH	•YouTube provides a significant content volume on LDH that widely ranges but is overall mediocre in quality. •Physician-directed educational videos were noted to be the only characteristic that reliably helps users identify a more useful information resource on YouTube.
Rudisill et al. [43]	To assess the reliability and educational quality of YouTube videos related to pediatric scoliosis	Searched using 4 different strings: “pediatric scoliosis”, “idiopathic scoliosis”, “scoliosis in children”, and “curved spine in children”; Selected first 50 videos in response to each query	153 •Based on the source: academic, physician (independent physician or group not affiliated with academic institutions), nonphysician (health professionals other than licensed medical doctors), athletic trainer, medical source (e.g., health website), patient (personal experience), and commercial. •Based on the content: rehabilitative exercises/physical therapy, disease-specific information, patient experience (personal accounts of disease and/or treatment), surgical management, nonsurgical management; and advertisement	JAMA score, GQS score, Pediatric Scoliosis-Specific Score	•YouTube contains a large repository of videos concerning pediatric scoliosis; however, the reliability and educational quality of these videos were low. •Multivariate analysis determined longer video duration predicted higher scores on all measures •There were no independent associations between upload source or content and assessment scores.
Richardson et al. [44]	To evaluate the quality and educational content of YouTube videos concerning spine tumors	Searched using keyword “spine tumor”; Selected first 50 videos in response to the query	50 •Based on the source: academic medical group, biomedical industry, private medical group, insurance company, and others. •Based on the content: advertisement, disease-specific information, nonsurgical management, patient experience, surgical technique or approach, and others.	JAMA score, GQS score, evaluation of specific information	•The reliability, quality, and educational content of YouTube videos were poor to suboptimal. •Videos that have been posted more recently attract more online attention •Video duration was positively associated with both the JAMA score and GQS; Number of views was associated with higher JAMA score; Number of dislikes was negatively associated with GQS.
Hornung et al. [24]	To characterize the educational quality and reliability of YouTube videos related to low back pain (LBP) as well as to identify factors associated with the overall video quality	Searched using 2 different strings: “low back pain” and “back pain”; Selected first 50 videos in response to each query	77 •Based on the source: academic (authors/uploaders with research or university/college affiliations), physician (physicians or physician groups without research or university/college affiliations), nonphysicians (health professionals other than licensed medical doctors), medical sources (content or animations from health-focused websites), and patients. •Based on the content: training (videos on exercise, rehabilitation, or therapy related to LBP), LBP-specific information (pathophysiology, testing, etc.), testimonials from patients, surgical technique(s), additional management procedures (nonsurgical or pharmacological), and advertisements	JAMA score, GQS score, LBP score (LPS)	•The overall reliability and educational quality of videos uploaded to YouTube concerning LBP are unsatisfactory. •More popular videos demonstrated poorer educational quality than their less popular counterparts. •Days since initial upload as well as like ratio were independent predictors of higher LPS scores.
Muller et al. [45]	To assess and compare the quality of lumbar fusion and arthroplasty videos on YouTube and to identify predictors of video quality	Searched using 6 different strings: “low back fusion”, “lumbar fusion”, “lumbar arthrodesis”, “low back disc replacement”, “lumbar disc replacement” and “lumbar disc arthroplasty”; Selected first 50 videos in response to each query	84 •Main category: educational, testimonial, commercial, and academic. •Subcategory: fusion (including different approach, e.g., anterior lumbar interbody fusion, transformainal lumbar interbody fusion, etc.), arthroplasty (including details of the implant, e.g., ProDisc-L, M6-L, etc.), non-specific.	JAMA score, informative score, clinical score	•Information on YouTube for lumbar fusion and arthroplasty is poor. •Information on fusion is better than arthroplasty. •Newer fusion videos had higher JAMA scores and fusion videos appearing sooner in search results had higher clinical scores; Longer fusion and arthroplasty videos both had higher clinical scores.
Stogowski et al. [38]	To assess the quality of the online videos regarding anterior lumbar interbody fusion (ALIF)	Searched using 3 different strings: “anterior lumbar interbody fusion”, “ALIF”, and “ALIF surgery”; Selected first 50 videos in response to each query	24 •No classification	DISCERN score	•The overall quality of YouTube videos on ALIF remains poor. •Longer video duration increases its quality without simultaneous negative influence on its popularity.

*JAMA* Journal of American Medical Association, *GQS* global quality scale, *HON* Health on the Net, *LDH* lumbar disc herniation, *LBP* low back pain, *LPS* LBP score, *ALIF* anterior lumbar interbody fusion

The low reliability and quality of medical videos on YouTube may be due to the absence of an access and censorship system. [30, 46] This allows unqualified individuals to publish videos on medical topics, potentially spreading unprofessional and unsubstantiated information. Of note that some videos were titled with phrases such as “treat with exercises”, “no surgery”, “best treatment”, which appeals to patients’ psychological needs. These videos contained considerably subjective statements with bias and may propagate inappropriate perception about the disease to viewers. Meanwhile, the recent fast-food culture (FFC) in video industry leads uploaders to create short, fast-paced video products or split videos into series, controlling the duration of less than 10 min to cater viewers’ preference [47]. In our study, the median duration of all involved videos was 263.00 (511.00) seconds (Table 5), and 73.15% (79/108) of videos were within 10 min long. This trend may inevitably fragment the intact information presented in a single video, leaving viewers with inadequate and unsystematic concepts.

In terms of specific content, the included videos were quite homogenous and shared several common issues. Nearly half (43%) of the videos discussed CS-specific information (Fig. 3B). Most videos covered the basic nosogenesis and typical symptoms of CS, but generally lacked deeper differentiation between CR and CM, which differ in severity, course and interfering methods. Videos should elucidate the divergence of their manifestations and managements precisely, and highlight the urgence and importance of early diagnosis and surgical intervention for CM, alerting viewers for accurate self-evaluations. In addition, the majority of videos pertaining to surgical techniques merely broached the fundamental concept, while few furnished detailed elucidations of the indications and advantages of various surgical approaches, which is a major concern for some viewers. In our outpatients, we often encountered patients who insisted on minimally invasive surgery without considering the objective fact that if the operative range is enough for the thorough decompression. It is challenging to coordinate with these patients who have preconceived notions and expectations. Therefore, it is necessary to inculcate them with comprehensive, objective and evidenced information about disease from the outset.

Despite the generally low quality of videos, there were still some of high caliber. For example, “cervical myelopathy and cervical radiculopathy- Everything You Need To Know—Dr. Nabil Ebraheim” from “nabil ebraheim”, “Cervical Radiculopathy—Why do you hurt and what is the plan to get you better?” from “Armaghani Spine” and “Exercises for pinched nerve in the neck (Cervical Radiculopathy) and neck pain relief” from “Dr. Andrea Furlan” were the top three videos that showed distinguished performance under our evaluation system. Our analysis revealed that the high-reliability and high-quality of videos tend to coexist with each other, and associated with certain video characteristics. Apparently, video duration is a crucial factor for reliability and quality, with a significant positive regression among them (Table 9). This finding was supported by other research. [48–50] Longer running time allows for more comprehensive coverage of topics and provide more educational information. Videos from academic source had significant advantages in both reliability and educational quality (Tables 6 and 9). These videos were produced by educational experts or groups in the spine field with high academic literacy, and were primarily aimed at clinicians or medical students, who demand higher breadth and accuracy of content. The source of clinicians could also be considered as an independent predictor of high quality (Table 9). Clinicians possess extensive clinical experience that could better meet the needs of users and patients. As increased licensed doctors participate in We-Media and share their clinical experience online, they achieve another avenue of communication with patients and gain considerable popularity, which is commendable. However, videos from clinicians did not demonstrate superiority in terms of reliability, suggesting that clinicians may not place sufficient emphasis clarifying reference sources, copyrights and qualifications etc.

**Table 9:** Multiple linear regression analysis of correlations between video characteristics and VPI, JAMA, mDISCERN, GQS, NPSS scores

	Unstandardized β	SE	95% CI	Standardized β	*P value*
**VPI (*R***^**2**^** = 0.624)**					
Number of comments	0.583	0.105	0.374 – 0.791	0.549	< *0.001*
Uploader verification	122.362	56.786	9.579 – 235.144	0.213	*0.034*
Sum of subscribers (× 10^–3^)	-0.061	0.023	-0.107—-0.014	-0.272	*0.011*
Video content					
CS-specific information	Ref				
Exercise training	164.943	57.479	50.784 – 279.103	0.293	*0.005*
**JAMA (*R***^**2**^** = 0.526)**					
Video duration (× 10^–3^)	0.295	0.063	0.170 – 0.419	0.378	< *0.001*
Like ratio	0.045	0.012	0.021 – 0.070	0.290	< *0.001*
Uploader verification	0.597	0.189	0.221 – 0.973	0.270	*0.002*
Video source					
Medical source	Ref				
Academic	0.609	0.199	0.213 – 1.005	0.269	*0.003*
**mDISCERN (*R***^**2**^** = 0.549)**					
Video duration (× 10^–3^)	0.336	0.082	0.174 – 0.498	0.316	< *0.001*
Like ratio	0.063	0.016	0.031 – 0.095	0.295	< *0.001*
Video source					
Medical source	Ref				
Academic	0.923	0.254	0.420 – 1.426	0.299	< *0.001*
Trainer	-1.504	0.440	-2.377—-0.631	-0.247	*0.001*
**GQS (*R***^**2**^** = 0.689)**					
Video duration (× 10^–3^)	0.604	0.074	0.456 – 0.751	0.607	< *0.001*
Video source					
Medical source	Ref				
Academic	0.730	0.216	0.300 – 1.159	0.253	*0.001*
Clinician	0.689	0.214	0.263 – 1.114	0.233	*0.002*
Video content					
CS-specific information	Ref				
Exercise training	-0.653	0.278	-1.205—-0.101	-0.236	*0.021*
Advertisement	-0.668	0.251	-1.166—-0.169	-0.180	*0.009*
**CSSS (*R***^**2**^** = 0.673)**					
Video duration (× 10^–3^)	3.140	0.382	2.382 – 3.899	0.603	< *0.001*
Video source					
Medical source	Ref				
Academic	3.345	1.174	1.015 – 5.676	0.222	*0.005*
Clinician	3.112	1.113	0.902 – 5.322	0.201	*0.006*
Non-clinician	-2.412	1.052	-4.500—-0.324	-0.186	*0.024*
Trainer	-4.657	2.152	-8.931—-0.384	-0.156	*0.033*

*SE* standard error, *CI* confidence interval, *VPI* video power index, *JAMA* Journal of American Medical Association, *mDISCERN* modified DISCERN, *GQS* Global Quality Scale, *CSSS* Cervical-Spondylosis-Specific Scale

The VPI, i.e. video popularity, comprises two components: views and likes. [30] Spearman correlation analysis indicated that there was no significant relationship between VPI and reliability or quality (Table 7). A higher VPI was associated with a greater number of comments (Table 9), showing better engagement with the video. The source of trainer and the corresponding content of exercise training may lead to a higher VPI (Fig. 4, Tables 6 and 9). Unfortunately, both were not able to predict higher reliability and quality, or even poorer than other sorts (Tables 6 and 9). Those videos may easily gain the favor of viewers, but lack in-depth elaboration of CS. On the contrary, videos about medical management, including both surgical or non-surgical categories, had relative advantages in reliability and quality (Table 6). However, neither topic showed a correlation with higher VPI. The results revealed that videos with higher dissemination value did not receive commensurate levels of attention. To address the conflict between video popularity and quality, it needs deeper consideration on what indeed influence the VPI.

**Fig. 5:**
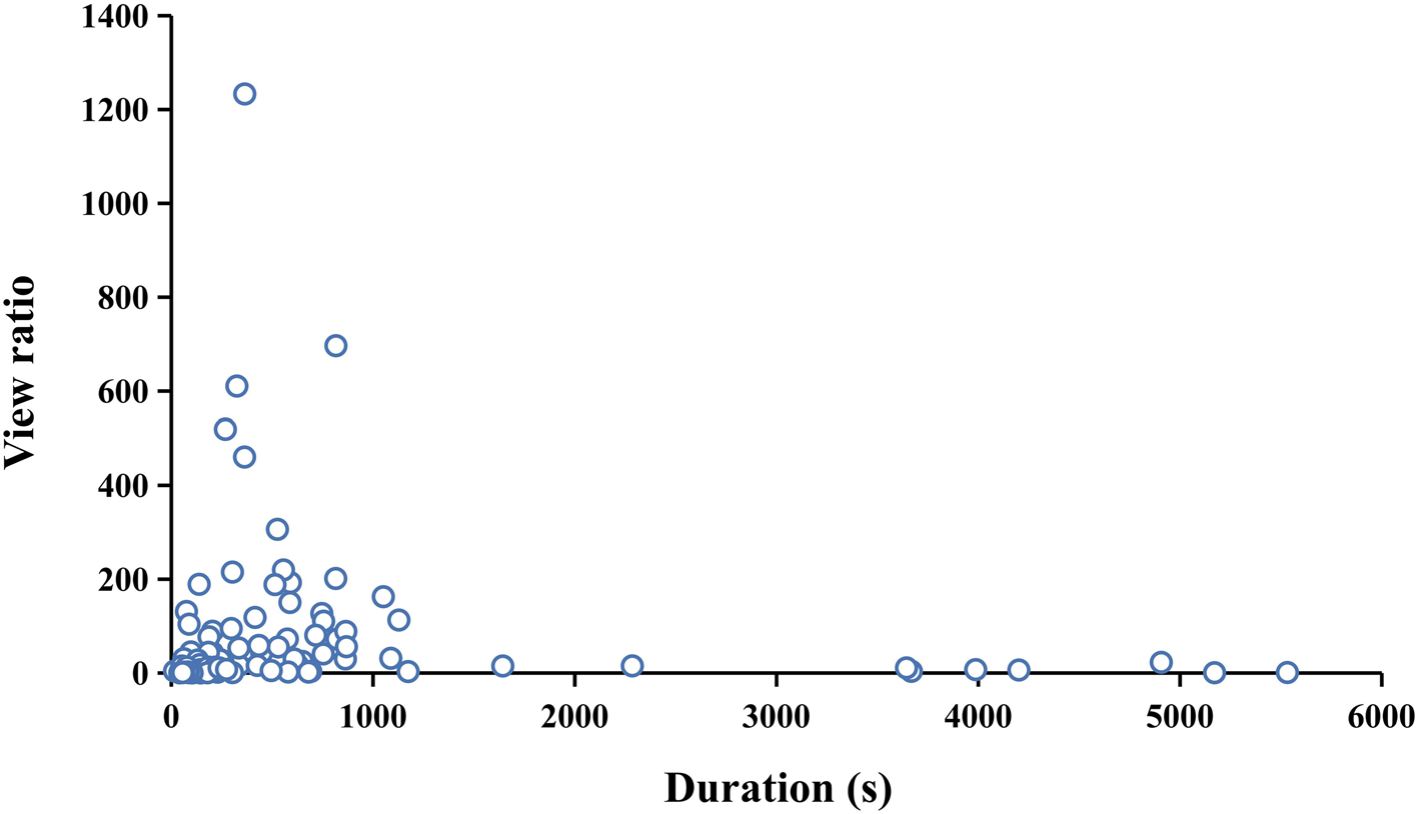
Distribution of duration and corresponding view ratio of each video

By deconstructing the VPI into two sub-indexes, we further focused on the view ratio. The diagram demonstrated that those shorter videos exceled in engaging viewers (Fig. 5), however, being short in quality, as mentioned above. This precisely reflected the so called FFC. While videos from high-caliber uploaders, like academics and clinicians, had relatively longer duration, occupying 33% of the video sum and 52.34% (36,863/70425) of total duration, but only 29.55% (3,569,505/12078971) of total views. These authorities or official channels often had lower rank and fewer subscribers. Undeniably, long videos tend to exert greater viewing pressure, occupying more real-life time and imposing amount of information to the audience to absorb, which may lead to potential resistance from users. In the era chasing for efficiency, the FFC which is naturally facilitated by viewers has its own rational grounds.

The simply short videos yet to have incontrovertible dominance to users that shorter duration didn’t independently predict a higher VPI (Table 9). And as another component of VPI, the importance of the like ratio should be emphasized. Notably, higher like ratio was independently associated with higher JAMA and mDISCERN scores, i.e., reliability (Table 9). This suggested that reliable and high-quality videos could ultimately gain viewers’ positive feedback. However, the viewing propensity are mainly determined by superficial and brief information on the index page, such as title, cover, duration and uploader etc. The specific content quality couldn’t exert direct influence on viewers’ choices. Meanwhile, as the ability to access, integrate and absorb online information varies and is generally limited when dealing with knowledge from other fields among lay users, the cognitions from them towards high-quality professional content are insensitive and unprecise. As Staunton et al. noted, higher-quality information may not always be in a readable manner to engage users. [51] A common problem currently plagues the high-quality videos is the poor comprehensibility that were too profound and lengthy for users to efficiently absorb the information. These prompts us there is potential and need to direct viewers from purely short and coarse videos to the high-quality videos with more reliable, concise and comprehensible information.

Enhancing the quality of internet medical information and optimizing the online patient education requires the concerted efforts of multiple parties. One solution to improve the overall reliability and educational quality of CS-related videos on YouTube may be to motivate academics and clinicians to produce more contents. As professionals, academics and clinicians should devote more effort on popularizing medical knowledge for the public. The dominance of short videos should be reconsidered. There is a need for deeper consideration of how to extract important, systematic knowledge from expertise and present it comprehensibly within a limited duration. Lightweight but not necessarily short videos could benefit to both gaining preference and delivering accurate information. Simultaneously, the professional videos could be wrapped and presented in an appropriate way that caters to the viewers’ mentality to direct them to the high-quality content more effectively. In another aspect, it is essential to strengthen the cognition of information reliability of the uploaders. The importance of unbiased and evidence-based information, copyright awareness and disclosure of interests etc. should be emphasized to engage users and, more importantly, to reduce the possibility of users being exploited by commercial interests. For the YouTube platform, advanced artificial intelligence could be used to establish more stringent admission requirements and screening systems to resist poor and misleading contents. Additionally, combining with big-data analysis, the platform could selectively promote and push high-quality videos and relevant evidence-based information to the target population. Last but not least, the public should improve their ability to obtain and utilize the internet information dialectically, striving to understand the underlying mechanisms and scientific managements of the diseases.

Our study had some unavoidable limitations. Given the popularity and clout, we only included English videos on YouTube as subjects, which may induce selective bias and reduce external validity. The quality scales, GQS and CSSS, were subjective and lacked strict validation. Although we adopted a double-review process, confounders were inevitable. Additionally, due to the inherent limitations of the scoring systems, our assessment was limited to the breadth of the covered topics in a video and did not evaluate the comprehensibility and efficiency of specific information delivering for the common users. It should also be mentioned that our statistics were limited by timeliness. Most characteristics data of videos changed dynamically and may not be representative for all periods. Besides, users may obtain more complete CS-related information from multiple complementary videos on YouTube. The results evaluated from each single video may not accurately quantify the holistic information that users could perceive from YouTube. More comprehensive evaluation methods remain to be explored. Beyond the scope of this study are other factors that may affect video popularity, such as potential social and commercial influences. Therefore, our findings about video popularity should be considered with caution.

## Conclusions

The internet has shown great potential in the medical field for patient education. However, the quality of online information is uneven and unregulated. This study indicated that the overall reliability and educational quality of current CS-related videos on YouTube are unsatisfactory. Users and patients searching for CS on YouTube are at high risk of encountering inadequate, inaccurate, or even misleading information. The videos with longer duration or from academic or clinician source could lead to higher reliability and quality. Optimizing the overall reliability and quality of online information requires a collaborative effort from multiple parties. We suggested motivating academics and clinicians to produce more concise and accurate contents. Meanwhile, the platform needs to establish stringent admission requirements and screening systems. And the public should endeavor to obtain and utilize internet information critically.

## Data Availability

The datasets used and/or analysed during the current study are available from the corresponding author on reasonable request.

## Abbreviations

CS: Cervical spondylosis
CR: Cervical radiculopathy
CM: Cervical myelopathy
VPI: Video Power Index
JAMA: Journal of American Medical Association
mDISCERN: Modified DISCERN
GQS: Global Quality Scale
CSSS: Cervical-Spondylosis-Specific Scale
FFC: Fast-food culture
