# Correction: YouTube online videos as a source for patient education of cervical spondylosis—a reliability and quality analysis

**DOI:** 10.1186/s12889-023-16807-0

**Published:** 2024-04-05

**Authors:** Wang Hong, Yan Chunyi, Wu Tingkui, Zhang Xiang, He Junbo, Liu Zhihao, Liu Hao

**Affiliations:** Department of Orthopedics, West China Hospital, Sichuan University, #37 Guoxue Alley, Wuhou District, Chengdu, Sichuan China

**Correction: BMC Public Health 23, 1831 (2023)**

**https://doi.org/10.1186/s12889-023-16495-w**

The original publication of this article [1] contained the redundant column “CSSS” in Table 9. The incorrect and correct version of table 9 are published in this correction article. The original article has been updated.

Incorrect version of Table 9 as originally published

**Table:** 

	Unstandardized β	SE	95% CI	Standardized β	*P value*	**CSSS**
**VPI (R**^**2**^** = 0.624)**						
Number of comments	0.583	0.105	0.374 – 0.791	0.549	< *0.001*	14.28 ± 5.65
Uploader verification	122.362	56.786	9.579 – 235.144	0.213	*0.034*	
Sum of subscribers (× 10^–3^)	-0.061	0.023	-0.107—-0.014	-0.272	*0.011*	
Video cozntent						8.30 ± 5.63
CS-specific information	Ref					
Exercise training	164.943	57.479	50.784 – 279.103	0.293	*0.005*	
**JAMA (R**^**2**^** = 0.526)**						
Video duration (× 10^–3^)	0.295	0.063	0.170 – 0.419	0.378	< *0.001*	
Like ratio	0.045	0.012	0.021 – 0.070	0.290	< *0.001*	
Uploader verification	0.597	0.189	0.221 – 0.973	0.270	*0.002*	
Video source						
Medical source	Ref					
Academic	0.609	0.199	0.213 – 1.005	0.269	*0.003*	
**mDISCERN (R**^**2**^** = 0.549)**						
Video duration (× 10^–3^)	0.336	0.082	0.174 – 0.498	0.316	< *0.001*	
Like ratio	0.063	0.016	0.031 – 0.095	0.295	< *0.001*	
Video source						
Medical source	Ref					
Academic	0.923	0.254	0.420 – 1.426	0.299	< *0.001*	
Trainer	-1.504	0.440	-2.377—-0.631	-0.247	*0.001*	
**GQS (R**^**2**^** = 0.689)**						
Video duration (× 10^–3^)	0.604	0.074	0.456 – 0.751	0.607	< *0.001*	
Video source						
Medical source	Ref					
Academic	0.730	0.216	0.300 – 1.159	0.253	*0.001*	
Clinician	0.689	0.214	0.263 – 1.114	0.233	*0.002*	
Video content						
CS-specific information	Ref					
Exercise training	-0.653	0.278	-1.205—-0.101	-0.236	*0.021*	
Advertisement	-0.668	0.251	-1.166—-0.169	-0.180	*0.009*	
**CSSS (R**^**2**^** = 0.673)**						
Video duration (× 10–3)	3.140	0.382	2.382 – 3.899	0.603	< *0.001*	
Video source						
Medical source	Ref					
Academic	3.345	1.174	1.015 – 5.676	0.222	*0.005*	
Clinician	3.112	1.113	0.902 – 5.322	0.201	*0.006*	
Non-clinician	-2.412	1.052	-4.500—-0.324	-0.186	*0.024*	
Trainer	-4.657	2.152	-8.931—-0.384	-0.156	*0.033*	
SE, standard error; CI, confidence interval; VPI, video power index; JAMA, Journal of American Medical Association; mDISCERN, modified DISCERN; GQS, Global Quality Scale; CSSS, Cervical-Spondylosis-Specific Scale						6.46 ± 2.87

Correct version of Table 9

**Table:** 

	Unstandardized β	SE	95% CI	Standardized β	*P value*
**VPI (R**^**2**^** = 0.624)**					
Number of comments	0.583	0.105	0.374 – 0.791	0.549	< *0.001*
Uploader verification	122.362	56.786	9.579 – 235.144	0.213	*0.034*
Sum of subscribers (× 10^–3^)	-0.061	0.023	-0.107 – -0.014	-0.272	*0.011*
Video content					
CS-specific information	Ref				
Exercise training	164.943	57.479	50.784 – 279.103	0.293	*0.005*
**JAMA (R**^**2**^** = 0.526)**					
Video duration (× 10^–3^)	0.295	0.063	0.170 – 0.419	0.378	< *0.001*
Like ratio	0.045	0.012	0.021 – 0.070	0.290	< *0.001*
Uploader verification	0.597	0.189	0.221 – 0.973	0.270	*0.002*
Video source					
Medical source	Ref				
Academic	0.609	0.199	0.213 – 1.005	0.269	*0.003*
**mDISCERN (R**^**2**^** = 0.549)**					
Video duration (× 10^–3^)	0.336	0.082	0.174 – 0.498	0.316	< *0.001*
Like ratio	0.063	0.016	0.031 – 0.095	0.295	< *0.001*
Video source					
Medical source	Ref				
Academic	0.923	0.254	0.420 – 1.426	0.299	< *0.001*
Trainer	-1.504	0.440	-2.377 – -0.631	-0.247	*0.001*
**GQS (R**^**2**^** = 0.689)**					
Video duration (× 10^–3^)	0.604	0.074	0.456 – 0.751	0.607	< *0.001*
Video source					
Medical source	Ref				
Academic	0.730	0.216	0.300 – 1.159	0.253	*0.001*
Clinician	0.689	0.214	0.263 – 1.114	0.233	*0.002*
Video content					
CS-specific information	Ref				
Exercise training	-0.653	0.278	-1.205 – -0.101	-0.236	*0.021*
Advertisement	-0.668	0.251	-1.166 – -0.169	-0.180	*0.009*
**CSSS (R**^**2**^** = 0.673)**					
Video duration (× 10–3)	3.140	0.382	2.382 – 3.899	0.603	< *0.001*
Video source					
Medical source	Ref				
Academic	3.345	1.174	1.015 – 5.676	0.222	*0.005*
Clinician	3.112	1.113	0.902 – 5.322	0.201	*0.006*
Non-clinician	-2.412	1.052	-4.500 – -0.324	-0.186	*0.024*
Trainer	-4.657	2.152	-8.931 – -0.384	-0.156	*0.033*

*SE* standard error, *CI* confidence interval, *VPI* video power index, *JAMA* Journal of American Medical Association, *mDISCERN* modified DISCERN, *GQS* Global Quality Scale, *CSSS* Cervical-Spondylosis-Specific Scale
